# Variation in tolerance to heterospecific pollen from a non‐native congener depends on co‐existence history of maternal and paternal source populations

**DOI:** 10.1002/ajb2.70139

**Published:** 2025-12-08

**Authors:** Yusuke Hoshino, Sachiko Horie, Masayuki Maki, Ikumi Dohzono

**Affiliations:** ^1^ Botanical Gardens Tohoku University, Kawauchi Sendai 980‐0862 Japan; ^2^ Department of Environmental Sciences Tokyo Gakugei University 4‐1‐1 Nukuikitamachi, Koganei Tokyo 184‐8501 Japan

**Keywords:** co‐flowering, heterospecific pollen, hybridization, interspecific pollen transfer, native, non‐native, *Oxalis*, tolerance

## Abstract

**Premise:**

Plants in sympatric populations with congeners may have evolved tolerance to the negative effects of heterospecific pollen (HP) through selection on female or male reproductive traits. If so, then the degree of HP tolerance may vary depending on the co‐existence history of the maternal and paternal plant source populations. Empirical evidence from species with a known history of contact is limited.

**Methods:**

This study focused on sympatric and allopatric populations of the native *Oxalis corniculata* and its recently arrived congener *O. dillenii* in Japan. Specifically, we conducted heterospecific and conspecific crosses with *O. corniculata* and two sequential pollination treatments using conspecific (CP) followed by heterospecific (HP) pollen, with CP donors from sympatric or allopatric populations.

**Results:**

Heterospecific crosses revealed lower seed production in *O. corniculata* than conspecific crosses, with sympatric populations showing a significantly greater reduction. Sequential pollination experiments reduced conspecific seed production, particularly when both the recipients and CP donor originated from sympatric populations.

**Conclusions:**

Our results suggest that individuals from sympatric populations may mitigate the negative effects of HP caused by hybrid seed formation. Furthermore, the variation in tolerance to the inhibition of conspecific fertilization by HP could be attributed to the recipient and CP donor origins. But the unexpectedly high susceptibility of sympatric populations to HP highlights the complex ecological and evolutionary factors that influence HP tolerance.

Heterospecific pollen (HP) transfer frequently occurs in co‐flowering plant communities due to shared pollinators (Feinsinger et al., 1986; Brown and Mitchell, 2001; Tur et al., 2016; Suárez‐Mariño et al., 2019). HP deposition may negatively affect the reproduction of flowering plants (Galen and Gregory, 1989; Celaya et al., 2015). In particular, HP deposition from closely related species may result in hybridization, resulting in the loss of ovules that would otherwise be fertilized with conspecific pollen (CP) (Burgess et al., 2008; Fukatsu et al., 2019). Moreover, HP can interfere with conspecific fertilization in several ways. For example, HP can physiologically interact with the stigma or CP grains through allelochemicals (pollen allelopathy) and, in some cases, interfere with CP fertilization (Murphy, 2000; Wilcock and Neiland, 2002). In addition, stylar clogging by HP may physically interfere with CP fertilization, consequently reducing seed set (Brown and Mitchell, 2001). A comparative analysis at the species level involving 20 pairs of plant species indicated that HP reduced average seed set by 20%, although the negative effects of HP varied widely among species pairs (Ashman and Arceo‐Gómez, 2013). Under constant HP deposition across years and the resultant negative impact on reproduction in plant species under natural conditions, natural selection may favor floral traits that can reduce HP deposition or ameliorate its deleterious effects on reproduction (Fang et al., 2019).

Modification of pre‐pollination processes can reduce HP deposition by segregating pollinators or avoiding pollen mixtures, such as divergence in flowering phenology (Borchsenius et al., 2016; Paudel et al., 2018), flower color (Hopkins and Rausher, 2012), floral scent (Hentrich et al., 2010), and pollen placement on the bodies of pollinators (Grant, 1994; Huang and Shi, 2013). Although these characteristics can minimize HP deposition, prepollination processes are not always sufficient to fully prevent HP transfer (Kay and Sargent, 2009; Moreira‐Hernandez et al., 2019). Therefore, plants are expected to develop tolerance mechanisms for HP that act at the post‐pollination stage and limit the inhibition of conspecific mating by HP (Moreira‐Hernandez et al., 2019; Suárez‐Mariño et al., 2019). However, post‐pollination tolerance to HP is not as well understood as pre‐pollination processes to avoid the negative effects of HP (Arceo‐Gómez et al., 2016; Fang et al., 2019).

Achieving HP tolerance is one of the expected evolutionary outcomes in flowering plants that co‐occur with congeners (Arceo‐Gómez et al., 2016; Fang et al., 2019; Moreira‐Hernandez et al., 2019). In this study, HP tolerance was defined as the capacity of a plant to endure continued exposure to pollen from other species without experiencing adverse effects on its reproductive success. For instance, *Costus pulverulentus* C.Presl (Costaceae) exhibits pollen–pistil incompatibility with HP from its congener *C. scaber* Ruiz & Pavon in sympatry, but not when the two species are allopatric (Kay and Schemske, 2008). This study indicated that increased tolerance to HP in maternal sporophytes has evolved over the history of co‐existence with congeners. In contrast, in *Clarkia xantiana* A.Gray (Onagraceae), adaptation to HP effects is suggested to occur in the male gametophytes, resulting in increased CP tube growth rates in sympatric populations with the co‐flowering congener *C. speciosa* F.H.Lewis & M.E.Lewis (Arceo‐Gómez et al., 2016). The results from these and other studies (Ashman and Arceo‐Gómez, 2013), collectively suggest that deleterious HP effects may be reduced through selection on either female or male traits. Accordingly, HP tolerance is expected to be lower when at least one parent in a conspecific cross lacks a history of co‐existence with a congener relative to the case where both parents have such a history. Therefore, experimental crosses using different combinations of sympatric and allopatric parents could provide a powerful approach for testing the hypothesis that HP tolerance evolves with exposure to pollen from congeners.

*Oxalis corniculata* L. (Oxalidaceae) is a self‐compatible perennial herb widely distributed in temperate and tropical regions. This species exhibits significant variation in the degree of herkogamy within and among populations, mostly because of the differences in pistil length (Shibaike et al., 1995; Hoshino et al., 2022). Homostyled plants with little herkogamy are capable of autonomously selfing at the end of anthesis (Shibaike et al., 1995; Hoshino et al., 2022). In contrast, long‐styled plants with a high degree of herkogamy avoid autonomous selfing, and their distribution is associated with high local pollinator availability (Hoshino et al., 2022). Although a floral morph polymorphism was observed, this variation did not constitute heterostyly, as it lacked reciprocal herkogamy. *Oxalis corniculata* can hybridize with *Oxalis dillenii* Jacq., which is native to North America, to form sterile hybrids in the wild (Fukatsu et al., 2019; Groom et al., 2021). However, hybrid seed formation is limited when the long‐styled plant of *O. corniculata* is the maternal parent, probably because the pollen of *O. dillenii* fails to fully elongate the pollen tubes (Hoshino et al., 2023). In Japan, the two *Oxalis* species coexist mainly in disturbed, human‐made habitats and exhibit substantial overlap during their flowering periods (Fukatsu et al., 2019; Hoshino and Dohzono, 2023). In addition, these species share small bee pollinators, *Halictus* spp. and *Lasioglossum* spp. (Halictidae) (Fukatsu et al., 2019), which may have resulted in the coexistence of HP on their stigmas. The suggestion that substantial HP transfer occurs between the two *Oxalis* species led us to hypothesize that *O. corniculata* populations experiencing long‐term co‐occurrence with *O. dillenii* have developed post‐pollination tolerance in response to the harmful effects of HP. In this study, we address the following questions (Figure 1): (1) If exposure to HP enables evolution of reproductive barriers, then hybrid seed set after HP pollination would be lower in sympatric than allopatric populations (Figure 1A); (2) If exposure to HP enables evolution of HP tolerance, then conspecific seed set after mixed pollinations would be higher in sympatry than in allopatry (Figure 1B); and (3) If exposure to HP enables evolution of HP tolerance via male function, then conspecific seed set would be lower when the paternal parent is from an allopatric population versus a sympatric population, and vice versa if via female function. (Figure 1C).

**Figure 1:**
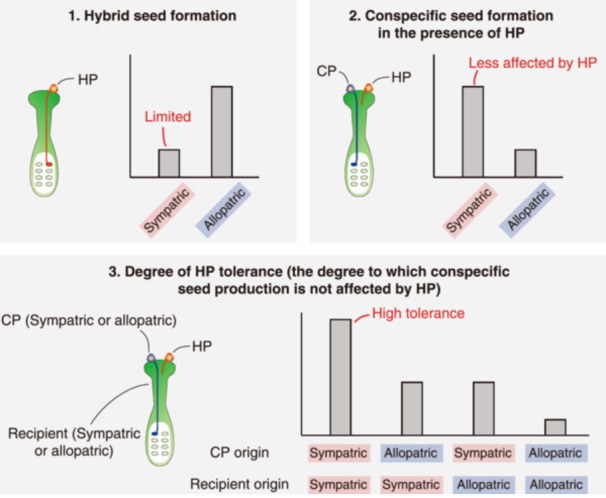
Conceptual diagram illustrating three expected outcomes if heterospecific pollen (HP) tolerance increases with exposure to heterospecific pollen. In sympatric populations, (1) hybrid seed formation frequency is expected to be limited, and (2) conspecific pollen (CP) fertilization is predicted to be less affected by HP receipt. Moreover, (3) the degree of HP tolerance (the extent to which conspecific seed production is not affected by HP) may be highest when both the CP donor and the recipient originate from sympatric populations.

## MATERIALS AND METHODS

### Study species and sites

*Oxalis corniculata* is widespread in temperate and warm regions worldwide (Amano, 2001). It is a native perennial weed found in various habitats in Japan, from disturbed grasslands to mountain ranges, and has a prolonged flowering period from April to October. The non‐native *O. dillenii* is a North American species naturalized in Europe and Japan (Groom et al., 2017; Fukatsu et al., 2019). The ancestral condition of *Oxalis* sect. *Corniculatae*, which includes *O. corniculata* and *O. dillenii*, may have been a tristylous mating system; however, these species are self‐compatible and have lost their tristyly (Ornduff, 1972). In Japan, *O. dillenii* only has homostyled plants (Fukatsu et al., 2019). Based on the specimen information available in the S‐net data portal (http://science-net.kahaku.go.jp/) and in Murata (1967), non‐native *O. dillenii* was estimated to have invaded Japan around the 1960s and has now expanded its range across almost all of Japan. While the native species can grow in dimly lit habitats, the non‐native *O. dillenii* prefers well‐lit environments and is naturalized mainly in disturbed habitats (Fukatsu et al., 2019). Non‐native *O. dillenii* peaks in flowering betweenMay and June and scarcely flowers during and after summer in Japan (Hoshino and Dohzono, 2023). Hybrid individuals with a morphology intermediate between the two *Oxalis* species were found in the disturbed habitat with their parental species (Fukatsu et al., 2019). These natural hybrids were F1 generations based on their chromosome number, nuclear DNA content, and apparent sterility, indicating that introgression is unlikely to occur (Fukatsu et al., 2019).

Five populations of *O. corniculata* from around Tokyo, Central Honshu, Japan, were used as the source populations in this study (Table 1). These populations were separated by at least 20 km. The populations DA1, HA1, and KOG were located in urban and suburban areas with well‐lit habitats. We refer to these as sympatric populations, in which both *Oxalis* species co‐occur with their hybrids (Hoshino and Dohzono, 2023). The allopatric populations IRI and OTK were located along forest road edges with occasionally dim habitats, where only *O. corniculata* was found (Hoshino and Dohzono, 2023). As a source of HP, we used *O. dillenii* from the KOG population for all recipients in each experiment. Plants from these populations were collected and cultivated in the laboratory under an 18 h light/6 h dark cycle and at approximately 23°C before using in the following experiments.

**Table 1:** Geographic information and sampling year for the five study sites. N: Number of *Oxalis corniculata* individuals sampled. At KOG, *Oxalis. dillenii* individuals were also sampled.

Population type	Code	Sampling locality	Latitude (N)	Longitude (E)	Altitude (m)	Sampling year
Sympatric	DA1	Daiba, Minato Ward, Tokyo Metro.	35.633	139.772	7.5	2022 (N = 39)
	HA1	Simoyamaguchi, Hayama Town, Kanagawa Pref.	35.258	139.579	6.3	2021 (N = 12), 2022 (N = 17)
	KOG	Nukuikitamachi, Koganei City, Tokyo Metro.	35.705	139.491	73.3	*O. corniculata*: 2015 (N = 24), 2018 (N = 40), 2021 (N = 6), 2022 (N = 5) *O. dillenii*: 2018 (N = 41), 2021 (N = 26), 2022 (N = 65)
Allopatric	IRI	Tozura, Okutama Town, Tokyo Metro.	35.809	139.004	954.9	2022 (N = 41)
	OTK	Sakaishimachibun, Hanno City, Saitama Pref.	35.903	139.231	243.6	2022 (N = 52)

### Conspecific and heterospecific crosses

To determine differences in the extent of HP fertilization in *O. corniculata* between allopatric and sympatric populations, heterospecific cross‐pollinations were conducted using plants from five populations. *O. corniculata* flowers were pollinated by *O. dillenii* pollen grains. Since HP from the KOG population was used in all experiments, HP was considered local or non‐local depending on the recipient population (for example, HP was considered local for KOG recipients and non‐local for the other populations). Experimental individuals were randomly selected from each population. Treatments were applied to 1–7 flowers for 16–32 plants per population. In total, *N* = 34 (DA1), 29 (HA1), 66 (KOG), 33 (IRI), and 47 (OTK) flowers were used. As a control, we also conducted conspecific crosses within each *O. corniculata* population using pollen from other individuals of the same population.Conspecific crosses were applied to 1–5 flowers for 15–28 plants per population. In total, *N* = 41 (DA1), 40 (HA1), 30 (KOG), 42 (IRI), and 3 (OTK) flowers were used. Before pollination, each recipient flower was emasculated and the absence of pollen grains on the stigma was confirmed using a stereomicroscope. All treated flowers were bagged and marked with a tag until they withered or the fruit fully matured. In these treatments, CP or HP was applied to ensure full coverage of the stigma. Approximately 90 or more pollen grains were confirmed to have adhered to the stigma. Each flower of *O. corniculata* and *O. dillenii* has approximately 39.5 ± 7.4 and 55.7 ± 13.2 ovules, respectively (Hoshino et al., unpublished data).

A generalized linear mixed model (GLMM; Poisson error, log‐link function) was used to investigate the effects of pollination treatment (conspecific or heterospecific), population type (allopatric or sympatric), and their interactions on the number of seeds per flower. Plant ID was included as a random factor. As previous studies have suggested the possibility of finescale local adaptation to the effects of HP (such as adaptation to specific genotypes of congeners within populations) (Kay and Schemske, 2008), we included HP origin (local or non‐local) as a covariate in the GLMM model. To conduct post‐hoc comparisons, we estimated the Bonferroni‐adjusted marginal means obtained through the *emmeans* function from the *emmeans* R package (Lenth, 2022). We used data on seed numbers following conspecific crosses in KOG from Hoshino et al. (2022) and data on seed numbers following both conspecific and heterospecific crosses in DA1 and HA1 from Hoshino et al. (2023). In both studies, *O. dillenii* from KOG was used as the HP source, which is consistent with the methods of this study.

Long‐styled *O. corniculata* plants may prevent fertilization by *O. dillenii* pollen (Hoshino et al., 2023). To examine this possibility, we extended the above GLMM model by including pistil length (scaled using z‐transformation with the *scale* function) as a covariate. Data were obtained from only four populations (DA1, HA1, IRI, and OTK). We excluded KOG, which included only homostyled plants with small variations in pistil length (Hoshino et al., 2022).By excluding KOG from the analysis, all HP sources were non‐local to the recipients. Therefore, HP origin was excluded as a covariate in this model. Pistil length was measured as described in Hoshino et al. (2022). Due to the inability to sample flowers for measurement, data from five individuals were excluded from the analysis.

### Sequential pollination experiments and conspecific seed production estimation

**Figure 2:**
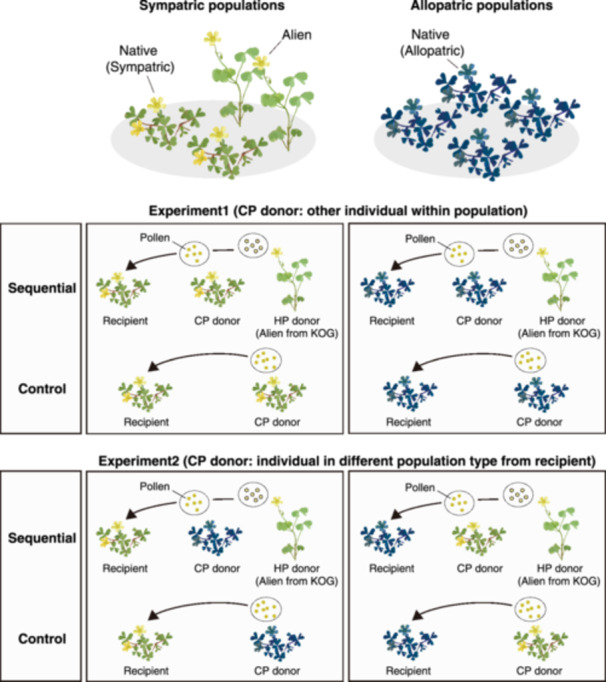
Experimental design of the two groups of pollination treatments (Experiment 1 and 2) on native *Oxalis corniculata* and non‐native *O. dillenii*. Each experiment includes sequential pollination treatment of conspecific and heterospecific pollen (donated as “CP” and “HP,” respectively) and corresponding control treatment utilizing only CP sources. The experiments differ in the origin of the CP donor utilized (sympatric or allopatric).

To investigate whether conspecific seed production of *O. corniculata* in sympatric populations is less likely to be hindered by HP than that in allopatric populations, sequential pollination treatments with CP and HP grains, along with the corresponding control treatments with only CP, were conducted. To test whether the degree of HP tolerance varied depending on the recipient and CP donor origin, we designed two treatments with CP donors of different origins (Experiments 1 and 2, Figure 2). In Experiment 1, local CP (CP from a different individual within the population) was used for both the control and sequential pollination treatments. Conversely, in Experiment 2, non‐local CP (CP derived from a different population type) was used for both control and sequential pollination (Figure 2). Consistent with the conspecific pollination described above, CP grains were applied to fully cover the stigma in both control and sequential pollination treatments. Immediately after CP application, HP from *O. dillenii* in KOG was applied, ensuring uniform surface coverage and forming a layer of HP over the CP in the sequential pollination treatments. Each pollination treatment was conducted on randomly selected 1 to 5 flowers per individual. In total, 6 to 28 individuals were used per population. In Experiment 2, the allopatric OTK population was used as CP donor for the recipient of the sympatric populations (DA1, HA1, and KOG) and the sympatric HA1 population was used for the recipient of the allopatric populations (IRI and OTK). All treated flowers were monitored for fruit development and the number of mature seeds per treated flower was counted. As a control treatment in Experiment 1, we used data on the number of seeds from the conspecific crosses conducted above. In sequential pollinations, the application of CP to the stigma before HP, should give CP a competitive advantage. However, because the effect of pollen arrival order was standardized across all populations, any differences in the relative effects of HP on conspecific seed production among population types were unlikely to attributable to this factor. Instead, such differences can be interpreted as reflecting variations in HP tolerance, that is, the extent to which conspecific seed production is maintained despite HP receipt.

To estimate the proportion of conspecific seeds produced in the sequential pollination treatments, seed paternity was estimated using PCR–RFLP analysis of the nuclear ribosomal ITS region. Seeds from 9 to 15 fruits per population were sown in Petri dishes with moistened filter paper and kept in a growth chamber under light conditions at 18°C for at least 35 d. Germinated seedlings were frozen at –20°C until DNA extraction. Total DNA was extracted using Plant DNA Isolation Reagent (Takara Bio Inc., Shiga, Japan). The nuclear ribosomal ITS region was PCR‐amplified with the primer set ITS‐4/ITS‐5 (White et al., 1990) according to the manufacturer's protocol, using the AmpliTaq Gold 360 Master Mix (Applied Biosystems, Waltham, Massachusetts, USA). The PCR products were digested with the restriction enzyme *sty*I (Thermo Scientific, Waltham, Massachusetts, USA) to exclude *O. corniculata* and hybrids of the two *Oxalis* species (Fukatsu et al., 2019). The restriction fragments were separated on a 1.0% agarose gel and stained using GelRed™ (Biotium, Fremont, California, USA) in TAE buffer. A total of 629 plants from all populations (39–90 plants per population) were genotyped.

Variation in the pistil length of *O. corniculata*, accompanied by variation in the HP fertilization rate (Hoshino et al., 2023), may have influenced the number of ovules capable of being fertilized by CP in sequential pollination treatments. Consequently, variation in pistil length among populations could mask the effect of population type on the proportion of conspecific seeds per fruit. Therefore, we tested whether the pistil length of *O. corniculata* affected the proportion of conspecific seeds per fruit after sequential pollination treatment using a GLMM (binomial error, logit‐link function). As in the conspecific and heterospecific cross‐experiments, we used data from only four populations (DA1, HA1, IRI, and OTK). In the GLMM, the ratio of conspecific seeds to the total number of genetically analyzed seeds per fruit and pistil length (scaled using the z‐transformation with the *scale* function) were included as the response variable and fixed effect, respectively. Plant ID was also included as a random factor.

Subsequently, we estimated the number of conspecific seeds per fruit after each sequential pollination treatment by multiplying the ratio of conspecific seeds to the total number of genetically analyzed seeds by the overall number of seeds formed per fruit. For fruits that were not subjected to PCR–RFLP, the number of conspecific seeds was estimated by multiplying the total number of seeds per fruit by the average ratio of conspecific seeds per fruit in the same population. To evaluate the effects of recipient and CP donor origins on conspecific seed production, we constructed a GLMM (Poisson error, log‐link function) for conspecific seed production, with pollination treatment (control or sequential) and recipient and CP donor origin (sympatric or allopatric) as explanatory variables. We also included HP origin (local or non‐local) and plant ID as covariates and random factors, respectively. To conduct post‐hoc comparisons, we estimated the Bonferroni‐adjusted marginal means obtained through the *emmeans* function from the *emmeans* R package (Lenth, 2022). All statistical analyses were performed using the R statistical package (version 4.3.3; R Core Team, 2024).

## RESULTS

### Conspecific and heterospecific crosses

**Figure 3:**
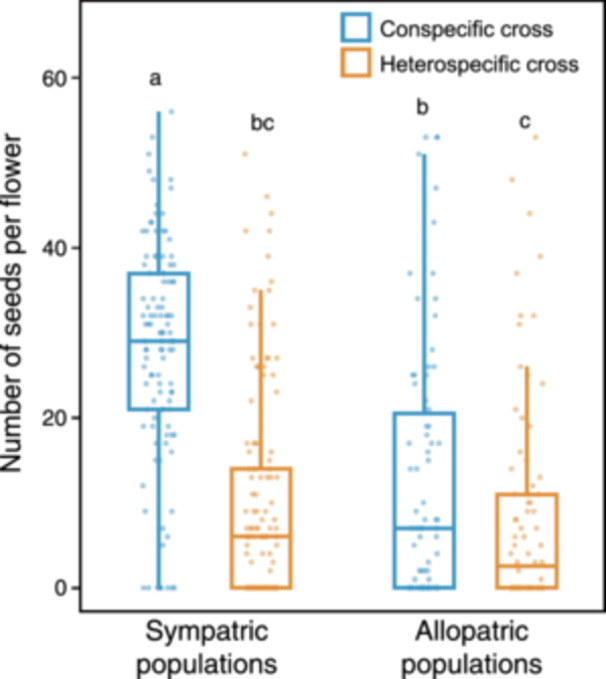
Seed production of *Oxalis corniculata* in three sympatric (DA1, HA1, and KOG) and two allopatric (IRI and OTK) populations with *O. dillenii* following conspecific and heterospecific crosses. Different letters indicate significant differences between the groups.

GLMM analysis revealed that pollination treatment, population type, and their interactions had significant effects on seed production of *O. corniculata* (Table 2). Heterospecific crosses resulted in lower seed production than did conspecific crosses in both sympatry and allopatry, but plants originating from allopatric populations produced fewer seeds than those originating from sympatric populations (Table 2, Figure 3). The reduction in seed production from conspecific to heterospecific crosses was greater in sympatric populations than in allopatric populations, as indicated by the significant interaction between pollination treatment and population type (Table 2, Figure 3). In contrast, the effect of HP origin was not significant (Table 2). The second GLMM, which included the pistil length of maternal *O. corniculata* individuals, revealed that pollination treatment, population type, and their interaction showed a significant effect on seed production (Appendices S1, S2). Although pistil length had a negative coefficient, the effect was not statistically significant (GLMM estimated coefficient = –0.25, SE = 0.15, *z* = –1.59, *P* = 0.111).

**Table 2:** Results of the generalized linear mixed models on seed production of *Oxalis corniculata* following the two pollination treatments (conspecific and heterospecific crosses).

Factor	Estimated coefficient	Std. error	*z*	*P*
Intercept (conspecific pollination, sympatric population)	2.98	0.20	14.66	**<0.001**
Pollination treatment (Pol)	–1.25	0.06	–21.09	**<0.001**
Population type (Pop)	–1.04	0.26	–4.01	**<0.001**
Heterospecific pollen origin	–0.02	0.28	–0.08	0.94
Pol × Pop	0.44	0.09	4.80	**<0.001**

### Sequential pollination and estimation of conspecific seed production

The total number of seeds per fruit resulting from sequential pollination in Experiments 1 and 2 varied from 16.8 in DA1 to 30.2 in KOG and from 11.9 in IRI to 18.1 in DA1 (Table 3). The average ratio of conspecific seeds to the total number of genetically analyzed seeds resulting from sequential pollination in Experiment 1 and 2 varied from 0.81 in KOG to 0.96 in HA1 and from 0.56 in OTK to 0.94 in IRI, respectively (Table 3). In 61% of the fruits, all genotyped seeds were identified as conspecific, whereas only 4.8% of the fruits contained exclusively hybrid seeds (Appendix S3). Pistil length of the recipients showed no significant effect on the ratio of conspecific seeds after the two sequential pollination treatments (GLMM estimated coefficient = –0.024, SE = 0.37, *z* = –0.064, *P* = 0.949) and was therefore dropped from subsequent analyses.

**Table 3:** Total number of seeds per fruit and ratio of conspecific seed following sequential pollination treatments in the two experiments (Experiments 1 and 2). See Figure 2 for details on the two experiments.

Population type	Code	Experiment 1 (CP donor: other individual within population)		Experiment 2 (CP donor: individual in different population type from recipient)	
		Total number of seed per fruit (mean ± SD)	Ratio of conspecific seed (mean ± SD)	Total number of seed per fruit (mean ± SD)	Ratio of conspecific seed (mean ± SD)
Sympatric	DA1	16.8 ± 15.0	0.93 ± 0.14	18.1 ± 13.8	0.92 ± 0.18
	HA1	21.1 ± 13.4	0.96 ± 0.079	13.4 ± 13.2	0.82 ± 0.32
	KOG	30.2 ± 18.9	0.81 ± 0.18	15.0 ± 13.8	0.83 ± 0.17
Allopatric	IRI	17.8 ± 14.3	0.92 ± 0.16	11.9 ± 14.9	0.94 ± 0.098
	OTK	22.0 ± 19.4	0.83 ± 0.21	14.7 ± 10.3	0.56 ± 0.44

**Table 4:** Results of generalized linear mixed model for the number of conspecific seeds resulting from the two sequential pollination experiments (Experiments 1 and 2). See Figure 2 for details on the two experiments.

Factor	Estimated coefficient	Std. error	z	*P*
Intercept (control treatment, recipient origin: sympatric, CP donor origin: sympatric)	3.28	0.13	26.1	**<0.001**
Pollination treatment (Pol)	–0.55	0.035	–15.45	**<0.001**
Recipient origin (Rep)	–0.74	0.16	–4.68	**<0.001**
CP donor origin (CP)	–0.25	0.028	–8.80	**<0.001**
HP donor origin	–0.27	0.17	–1.55	0.12
Pol × Rep	0.55	0.046	11.90	**<0.001**
Pol × CP	0.26	0.041	6.41	**<0.001**

**Figure 4:**
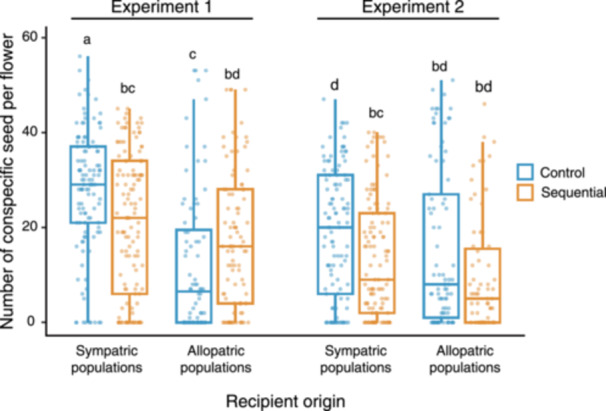
Conspecific seed production of *Oxalis corniculata* following sequential pollination with conspecific (CP) and heterospecific pollen (HP), and corresponding control treatments with CP only. Experiment 1 used local CP (from a different individual within the same population) for both control and sequential pollinations. Experiment 2 used non‐local CP (from a different population and population type than the recipient) for both control and sequential pollinations. Different letters denote significant differences among treatment groups.

The results of the two treatment groups with different CP donor origins (Experiments 1 and 2) demonstrated that pollination treatment had a significant effect on conspecific seed production (Table 4). In contrast, the effect of HP origin was not significant (Table 4). Overall, sequential pollination resulted in fewer conspecific seeds. We also found that conspecific seed production varied significantly between recipient and CP donor origins (Table 4). Conspecific seed production decreased when the crosses involved recipients and CP donors from allopatric populations (Table 4). We have also found a significant interaction between pollination treatment and the recipient or CP donor origin (Table 4). The reduction in conspecific seed production from the control to the sequential pollination was greater when the crossing involved recipients and CP donors from sympatric populations (Table 4). Posthoc comparisons further clarified the patterns of these interactions. Sequential pollination resulted in fewer conspecific seeds in recipients from sympatric populations than in the control in each experiment (Figure 4). In contrast, sequential pollination led to higher conspecific seed production among recipients from allopatric populations in Experiment 1, and a level of seed production comparable to that of the control in Experiment 2 (Figure 4).

## DISCUSSION

Generally, HP has negative impacts on reproduction in flowering plants (Brown and Mitchell, 2001), yet a history of co‐existence with congeners could lead to the selection for greater tolerance to HP effects (Ashman and Arceo‐Gómez, 2013; Arceo‐Gómez et al., 2016; Streher et al., 2020). We investigated whether *O. corniculata* populations that co‐occurred with *O. dillenii* evolved post‐pollination tolerance to mitigate the harmful effects of HP. We further hypothesized that the degree of HP tolerance may vary depending on the recipient and CP donor origin, based on the suggestion that deleterious HP effects can be reduced through selecting on either female or male reproductive traits (Arceo‐Gómez, 2021). Our results suggest that the negative effects of HP caused by hybrid seed formation are limited in sympatric populations. Additionally, the rate of conspecific seed production in the presence of HP varied depending on the recipient and CP donor origin; however, high HP tolerance was not consistently associated with a history of co‐existence with congeners. Therefore, our findings provide only partial support for the hypothesis that approximately 60 years of coexistence with a non‐native congener has promoted the evolution of HP tolerance in native *O. corniculata*. Non‐native plant species can impose strong selective pressures on natives (Callaway et al., 2005; Gibson et al., 2018), sometimes leading to rapid evolutionary responses within only 20 to 30 years (Callaway et al., 2005). However, the timescale over which HP tolerance evolves remains largely unknown; to date, only one study has reported stronger HP tolerance in populations with more than 30 years of co‐existence with a congener (Arceo‐Gómez et al., 2016). These mixed results underscore the need for cautious interpretation of our results, considering ecological and evolutionary factors beyond coexistence history.

Native *O. corniculata* can hybridize with non‐native *O. dillenii* to form sterile hybrids, which results in a waste of gametes and may cause reproductive interference between the two parental species (Fukatsu et al., 2019; Groom et al., 2021). Our results of the fewer total number of seeds with heterospecific crosses than conspecific crosses indicated that *O. corniculata* can produce hybrid seeds; however, it exhibited incomplete genetic compatibility with *O. dillenii* across all populations (Table 2, Figure 3). Additionally, we detected a significant interaction between pollination treatment and population type, regardless of whether the GLMM included pistil length as a factor (Table 2). This result suggests that the relative negative HP effects on reproduction are more limited in sympatric populations than in allopatric populations. Specifically, individuals in sympatric populations may have reproductive advantages by reducing resource losses due to hybrid seed formation, thereby preserving resources for conspecific offspring. Additionally, this may ameliorate the competitive costs of sharing pollinators with sympatric relatives (Moreira‐Hernández et al., 2023).

The non‐significant effect of HP origin suggests that *O. corniculata* lacks the capacity for fine‐scale local adaptation to congeners (Table 2), consistent with the findings of a previous study (Arceo‐Gómez et al., 2016). However, because of the limited replication of the HP origin in this experiment, the results should be viewed as preliminary and further investigations involving multiple sources of both local and non‐local HP are needed. In addition, we are currently unable to provide a clear explanation for the differences in seed production among population types, particularly those observed during conspecific pollination (Figure 3). Although such variability could be influenced by environmental heterogeneity such as variations in water availability, soil pH, and resources (Pérez‐Ramos et al., 2014; Recart et al., 2019), our current data did not allow us to test these possibilities directly. Further research is required to investigate the relationship between seed production and environmental factors at each site.

Sequential pollination treatments revealed that HP may have a negative effect on conspecific seed production (Table 4). Since the CP grains were applied in the same manner for both treatments, the reduction in conspecific seed production is likely not primarily due to the difference in the amount of applied CP grains but rather to the presence or absence of HP. The allelopathic effects of HP reported in several studies, such as interference with CP germination or CP tube growth, may explain the reduction in conspecific seed production (Kanchan and Chandra, 1980; Murphy, 2000). Additionally, we cannot exclude other factors that may affect conspecific seed production, such as stylar clogging (Brown and Mitchell, 2001). Since HP application after CP formed a certain number of hybrid seeds, it is possible that the pollen tubes of HP physically overcrowded the stylar tissue, leading to a lower CP fertilization rate. Furthermore, HP may induce interspecific seed discounting that may lead to seed abortion (Burgess et al., 2008; Montgomery et al., 2010). These possibilities may be verified by examining the dynamics of in vivo pollen tube growth after both control and sequential pollination treatments.

The effect of HP on conspecific seed production was varied depending on both the recipient and CP donor origins (Table 4). This result was predicted based on the view that HP may interact with both CP grains and the stigma itself on the stigmatic surface (Ashman and Arceo‐Gómez, 2013; Moreira‐Hernández and Muchhala, 2019), thereby exerting selective pressures on both female and male traits to counteract the negative effects of HP. However, contrary to our expectations, individuals from sympatric populations were more susceptible to the inhibition of conspecific fertilization by HP, whereas individuals from allopatric populations demonstrated higher tolerance, as evidenced by less reduction in conspecific seed production after HP exposure (Figure 4). It should be noted that the negative effects of HP may have been underestimated in our sequential pollination treatments because of the advantage conferred by the earlier arrival of CP on stigma. Accordingly, individuals from allopatric populations cannot demonstrate complete tolerance to HP.

Nevertheless, there are several possible explanations for this unexpected outcome that can be applied to our system. First, it is possible that species other than *O. dillenii* in each community affect HP tolerance of *O. corniculata*, as even HP from distantly related species can reduce reproductive success (Thomson et al., 1982; Lanuza et al., 2021). In this case, HP tolerance may evolve in response to selective pressures from the most abundant and harmful species, and tolerance may be detected independent of the sympatry with *O. dillenii*. However, because the negative effect of HP on conspecific mating is often greater when HP originates from closely related species (Streher et al., 2020), whether HP from distantly related species exerts selection for greater tolerance to HP remains unclear.

Second, the difference in the local pollination environments may have affected conspecific seed production. For example, pollen from outcrossing populations often shows better performance than pollen from predominantly selfing populations (Smith‐Huerta, 1997; Mazer et al., 2010), which may confer a competitive advantage against HP. Indeed, *O. corniculata* exhibits variations in herkogamy, suggesting a range of outcrossing rates (Shibaike et al., 1995). However, the allopatric populations that exhibited a higher HP tolerance in the present study did not necessarily display a greater degree of herkogamy than the sympatric populations (Hoshino et al., 2022).

Alternatively, individuals in sympatric populations may actively limit seed production, including conspecific seeds, when they received HP to avoid wasting resources on production of inviable hybrids. Resource allocation for reproduction may affect subsequent reproduction or growth in many iteroparous (organisms that are typically long‐lived and capable of reproducing multiple times) species (Newell, 1991; Obeso, 2002). Therefore, the temporary suppression of seed production in flowers that accidentally received a high amount of HP may confer advantages for subsequent reproduction, in other flowers within the inflorescence, within the same individual, or in the following year. Conversely, individuals in allopatric populations might not have decreased seed production, however, they produced a substantial number of hybrid seeds, resulting in an overall decrease in fitness. This hypothesis, which proposes new mechanisms of HP tolerance, can be validated by tracking long‐term seed production and vegetative growth of *O. corniculata* from each population type.

## CONCLUSIONS

This study revealed that *O. corniculata* from populations sympatric with the non‐native congener *O. dillenii* may mitigate the negative reproductive effects associated with hybrid seed formation. Moreover, using PCR‐RFLP analysis, we showed that HP negatively affects conspecific seed production. The negative HP effect varied depending on the origins of both recipient and CP donors, suggesting that the evolutionary histories of female and male traits, and that interactions may influence the degree of HP tolerance. This intraspecific variation in HP tolerance provides important insights into the evolutionary potential of flowering plants in response to HP deposition. Although HP deposition can be a strong factor that imposes selective pressure promoting the evolution of tolerance to its negative effects, high tolerance has not been consistently associated with a history of co‐existence with congeners. This suggests that environmental factors other than sympatric co‐existence with congeners may also contribute to shaping the degree of HP tolerance, thereby underscoring the complexity of the ecological and evolutionary factors involved. It should also be noted that the present study did not assess pre‐pollination adaptation to HP. Future research should address this aspect to gain a more comprehensive understanding of the significance of post‐pollination responses to HP revealed in this study, as well as the mechanisms underlying the coexistence of the two *Oxalis* species.

## AUTHOR CONTRIBUTIONS

Y.H. conceived and designed the experiments; Y.H. and I.D. conducted fieldwork; S.H. and M.M. developed methodology of molecular analyses. Y.H. and S.H. performed the laboratory experiments; and Y.H. drafted the manuscript. All authors contributed to the interpretation of the results and the writing.

## Supporting information


**Appendix S1.** The relationship between seed production following two pollination treatments (conspecific and heterospecific crosses) and pistil length of *Oxalis corniculata* in two sympatric (DA1 and HA1) and two allopatric populations (IRI and OTK).

**Appendix S2.** Results of the generalized linear mixed models on seed production of *Oxalis corniculata* following the two pollination treatments (conspecific and heterospecific crosses) in two sympatric (DA1 and HA1) and two allopatric populations (IRI and OTK).

**Appendix S3.** Results of PCR‐RFLP analysis for progenies of *Oxalis corniculata* following sequential pollination treatment in two experiments (Experiment 1 and Experiment 2). See Figure 2 for details on the two experiments.

## Data Availability

Data are available at Zenodo: https://doi.org/10.5281/zenodo.17271527.
