# Supplementary material for: Variation in tolerance to heterospecific pollen from a non‐native congener depends on co‐existence history of maternal and paternal source populations
Source: Am J Bot. 2025 Dec 8;112(12):e70139. doi: 10.1002/ajb2.70139 (PMC12712778; doi:10.1002/ajb2.70139)
Supplement: Supplementary file 1 — Appendix S1. The relationship between seed production following two pollination treatments (conspecific and heterospecific crosses) and pistil length of Oxalis corniculata in two sympatric (DA1 and HA1) and two allopatric populations (IRI and OTK). [file AJB2-112-e70139-s001.docx]

Appendix S1. The relationship between seed production following two pollination treatments (conspecific and heterospecific crosses) and pistil length of *Oxalis corniculata* in two sympatric (DA1 and HA1) and two allopatric populations (IRI and OTK).
