# Supplementary material for: Variation in tolerance to heterospecific pollen from a non‐native congener depends on co‐existence history of maternal and paternal source populations
Source: Am J Bot. 2025 Dec 8;112(12):e70139. doi: 10.1002/ajb2.70139 (PMC12712778; doi:10.1002/ajb2.70139)
Supplement: Supplementary file 2 — Appendix S2. Results of the generalized linear mixed models on seed production of Oxalis corniculata following the two pollination treatments (conspecific and heterospecific crosses) in two sympatric (DA1 and HA1) and two allopatric populations (IRI and OTK). [file AJB2-112-e70139-s002.docx]

| Appendix S2. Results of the generalized linear mixed models on seed production of *Oxalis corniculata* following the two pollination treatments (conspecific and heterospecific crosses). Data were obtained from two sympatric populations (DA1 and HA1) and two allopatric populations (IRI and OTK) that exhibit variation in pistil length. | | | | |
| --- | --- | --- | --- | --- |
| Factor | Estimated coefficient | Std. error | *z* | *p* |
| Intercept | 3.01 | 0.21 | 14.64 | **< 0.001** |
| Pollination treatment (Pol) | -1.24 | 0.06 | -20.79 | **< 0.001** |
| Population type (Pop) | -1.11 | 0.28 | -4.01 | **< 0.001** |
| Pistil length | -0.25 | 0.15 | -1.59 | 0.11 |
| Pol × Pop | 0.38 | 0.096 | 3.99 | **< 0.001** |
