# Supplementary material for: Variation in tolerance to heterospecific pollen from a non‐native congener depends on co‐existence history of maternal and paternal source populations
Source: Am J Bot. 2025 Dec 8;112(12):e70139. doi: 10.1002/ajb2.70139 (PMC12712778; doi:10.1002/ajb2.70139)
Supplement: Supplementary file 3 — Appendix S3. Results of PCR‐RFLP analysis for progenies of Oxalis corniculata following sequential pollination treatment in two experiments (Experiment 1 and Experiment 2). See Figure 2 for details on the two experiments. [file AJB2-112-e70139-s003.docx]

| Appendix S3. Results of PCR-RFLP analysis for progenies of *Oxalis corniculata* following mixed pollination treatment in two experiments (Experiment 1 and Experiment 2). See Figure 2 for details on the two experiments. | | | | | | | |
| --- | --- | --- | --- | --- | --- | --- | --- |
|  | Population | Plant ID | Fruit ID | Total number of seeds genotyped | Number of conspecific seeds | Number of hybrid seeds | Conspecific seed ratio |
| Experiment 1 | DA1 | D1 | exp1-D1-1 | 7 | 5 | 2 | 0.7142857 |
|  |  | D2 | exp1-D2-1 | 6 | 6 | 0 | 1 |
|  |  | D3 | exp1-D3-1 | 6 | 6 | 0 | 1 |
|  |  | D4 | exp1-D4-1 | 6 | 6 | 0 | 1 |
|  |  | D5 | exp1-D5-1 | 6 | 4 | 2 | 0.6666667 |
|  |  | D6 | exp1-D6-1 | 5 | 5 | 0 | 1 |
|  |  | D7 | exp1-D7-1 | 10 | 10 | 0 | 1 |
|  |  | D7 | exp1-D7-2 | 8 | 8 | 0 | 1 |
|  |  | D8 | exp1-D8-1 | 3 | 3 | 0 | 1 |
|  | HA1 | H1 | exp1-H1-1 | 10 | 10 | 0 | 1 |
|  |  | H2 | exp1-H2-1 | 9 | 7 | 2 | 0.7777778 |
|  |  | H3 | exp1-H3-1 | 7 | 7 | 0 | 1 |
|  |  | H4 | exp1-H4-1 | 6 | 6 | 0 | 1 |
|  |  | H5 | exp1-H5-1 | 11 | 11 | 0 | 1 |
|  |  | H6 | exp1-H6-1 | 7 | 6 | 1 | 0.8571429 |
|  |  | H7 | exp1-H7-1 | 5 | 5 | 0 | 1 |
|  |  | H8 | exp1-H8-1 | 2 | 2 | 0 | 1 |
|  |  | H9 | exp1-H9-1 | 7 | 7 | 0 | 1 |
|  |  | H10 | exp1-H10-1 | 6 | 6 | 0 | 1 |
|  | KOG | K1 | exp1-K1-1 | 21 | 18 | 3 | 0.8571429 |
|  |  | K2 | exp1-K2-1 | 16 | 14 | 2 | 0.875 |
|  |  | K3 | exp1-K3-1 | 2 | 2 | 0 | 1 |
|  |  | K4 | exp1-K4-1 | 6 | 3 | 3 | 0.5 |
|  |  | K5 | exp1-K5-1 | 14 | 12 | 2 | 0.8571429 |
|  |  | K6 | exp1-K6-1 | 7 | 5 | 2 | 0.7142857 |
|  |  | K7 | exp1-K7-1 | 3 | 3 | 0 | 1 |
|  |  | K8 | exp1-K8-1 | 11 | 8 | 3 | 0.7272727 |
|  |  | K9 | exp1-K9-1 | 10 | 9 | 1 | 0.9 |
|  | IRI | I1 | exp1-I1-1 | 2 | 2 | 0 | 1 |
|  |  | I2 | exp1-I2-1 | 5 | 5 | 0 | 1 |
|  |  | I3 | exp1-I3-1 | 5 | 4 | 1 | 0.8 |
|  |  | I4 | exp1-I4-1 | 3 | 3 | 0 | 1 |
|  |  | I5 | exp1-I5-1 | 10 | 10 | 0 | 1 |
|  |  | I6 | exp1-I6-1 | 4 | 2 | 2 | 0.5 |
|  |  | I7 | exp1-I7-1 | 7 | 5 | 2 | 0.7142857 |
|  |  | I8 | exp1-I8-1 | 4 | 4 | 0 | 1 |
|  |  | I9 | exp1-I9-1 | 10 | 10 | 0 | 1 |
|  |  | I10 | exp1-I10-1 | 3 | 3 | 0 | 1 |
|  |  | I11 | exp1-I11-1 | 5 | 5 | 0 | 1 |
|  |  | I12 | exp1-I12-1 | 2 | 2 | 0 | 1 |
|  | OTK | O1 | exp1-O1-1 | 7 | 6 | 1 | 0.8571429 |
|  |  | O2 | exp1-O2-1 | 5 | 5 | 0 | 1 |
|  |  | O3 | exp1-O3-1 | 14 | 13 | 1 | 0.9285714 |
|  |  | O4 | exp1-O4-1 | 3 | 3 | 0 | 1 |
|  |  | O4 | exp1-O4-2 | 15 | 15 | 0 | 1 |
|  |  | O5 | exp1-O5-1 | 17 | 9 | 8 | 0.5294118 |
|  |  | O6 | exp1-O6-1 | 4 | 3 | 1 | 0.75 |
|  |  | O7 | exp1-O7-1 | 8 | 4 | 4 | 0.5 |
|  |  | O7 | exp1-O7-1 | 3 | 3 | 0 | 1 |
|  |  | O9 | exp1-O9-1 | 7 | 5 | 2 | 0.7142857 |
|  |  | O10 | exp1-O10-1 | 2 | 1 | 1 | 0.5 |
|  |  | O11 | exp1-O11-1 | 3 | 3 | 0 | 1 |
|  |  | O10 | exp1-O10-2 | 2 | 2 | 0 | 1 |
| Experiment 2 | DA1 | D9 | exp2-D9-1 | 13 | 13 | 0 | 1 |
|  |  | D3 | exp2-D3-1 | 6 | 6 | 0 | 1 |
|  |  | D10 | exp2-D10-1 | 2 | 2 | 0 | 1 |
|  |  | D11 | exp2-D11-1 | 4 | 4 | 0 | 1 |
|  |  | D12 | exp2-D12-1 | 7 | 7 | 0 | 1 |
|  |  | D7 | exp2-D7-1 | 5 | 5 | 0 | 1 |
|  |  | D13 | exp2-D13-1 | 4 | 2 | 2 | 0.5 |
|  |  | D6 | exp2-D6-1 | 2 | 2 | 0 | 1 |
|  |  | D14 | exp2-D14-1 | 5 | 4 | 1 | 0.8 |
|  | HA1 | H11 | exp2-H11-1 | 4 | 4 | 0 | 1 |
|  |  | H12 | exp2-H12-1 | 9 | 9 | 0 | 1 |
|  |  | H6 | exp2-H6-1 | 9 | 9 | 0 | 1 |
|  |  | H13 | exp2-H13-1 | 6 | 6 | 0 | 1 |
|  |  | H2 | exp2-H2-1 | 6 | 6 | 0 | 1 |
|  |  | H14 | exp2-H14-1 | 4 | 4 | 0 | 1 |
|  |  | H16 | exp2-H16-1 | 5 | 3 | 2 | 0.6 |
|  |  | H15 | exp2-H15-1 | 4 | 3 | 1 | 0.75 |
|  |  | H8 | exp2-H8-1 | 4 | 0 | 4 | 0 |
|  |  | H16 | exp2-H16-2 | 5 | 4 | 1 | 0.8 |
|  | KOG | K10 | exp2-K10-1 | 4 | 4 | 0 | 1 |
|  |  | K11 | exp2-K11-1 | 5 | 5 | 0 | 1 |
|  |  | K10 | exp2-K10-2 | 4 | 4 | 0 | 1 |
|  |  | K12 | exp2-K12-1 | 3 | 3 | 0 | 1 |
|  |  | K13 | exp2-K13-1 | 6 | 6 | 0 | 1 |
|  |  | K10 | exp2-K10-3 | 8 | 7 | 1 | 0.875 |
|  |  | K12 | exp2-K12-2 | 3 | 3 | 0 | 1 |
|  |  | K14 | exp2-K14-1 | 2 | 1 | 1 | 0.5 |
|  |  | K15 | exp2-K15-1 | 4 | 4 | 0 | 1 |
|  | IRI | I13 | exp2-I13-1 | 16 | 15 | 1 | 0.9375 |
|  |  | I6 | exp2-I6-1 | 4 | 4 | 0 | 1 |
|  |  | I14 | exp2-I14-1 | 5 | 5 | 0 | 1 |
|  |  | I8 | exp2-I8-1 | 4 | 4 | 0 | 1 |
|  |  | I3 | exp2-I3-1 | 2 | 2 | 0 | 1 |
|  |  | I12 | exp2-I12-1 | 10 | 8 | 2 | 0.8 |
|  |  | I11 | exp2-I11-1 | 1 | 1 | 0 | 1 |
|  |  | I15 | exp2-I15-1 | 4 | 3 | 1 | 0.75 |
|  |  | I16 | exp2-I16-1 | 2 | 2 | 0 | 1 |
|  | OTK | O12 | exp2-O12-1 | 7 | 7 | 0 | 1 |
|  |  | O13 | exp2-O13-1 | 2 | 2 | 0 | 1 |
|  |  | O14 | exp2-O14-1 | 6 | 4 | 2 | 0.6666667 |
|  |  | O10 | exp2-O10-1 | 7 | 0 | 7 | 0 |
|  |  | O7 | exp2-O7-1 | 4 | 1 | 3 | 0.25 |
|  |  | O15 | exp2-O15-1 | 5 | 2 | 3 | 0.4 |
|  |  | O16 | exp2-O16-1 | 5 | 5 | 0 | 1 |
|  |  | O17 | exp2-O17-1 | 4 | 0 | 4 | 0 |
|  |  | O18 | exp2-O18-1 | 4 | 4 | 0 | 1 |
|  |  | O19 | exp2-O19-1 | 4 | 4 | 0 | 1 |
|  |  | O6 | exp2-O6-1 | 2 | 2 | 0 | 1 |
|  |  | O18 | exp2-O18-2 | 3 | 1 | 2 | 0.3333333 |
|  |  | O14 | exp2-O14-2 | 7 | 0 | 7 | 0 |
|  |  | O15 | exp2-O15-2 | 6 | 0 | 6 | 0 |
|  |  | O9 | exp2-O9-1 | 5 | 4 | 1 | 0.8 |
